# Glucagon-Like Peptide-1 Receptor Agonist Protects Dorsal Root Ganglion Neurons against Oxidative Insult

**DOI:** 10.1155/2019/9426014

**Published:** 2019-02-21

**Authors:** Mohammad Sarif Mohiuddin, Tatsuhito Himeno, Rieko Inoue, Emiri Miura-Yura, Yuichiro Yamada, Hiromi Nakai-Shimoda, Saeko Asano, Makoto Kato, Mikio Motegi, Masaki Kondo, Yusuke Seino, Shin Tsunekawa, Yoshiro Kato, Atsushi Suzuki, Keiko Naruse, Koichi Kato, Jiro Nakamura, Hideki Kamiya

**Affiliations:** ^1^Division of Diabetes, Department of Internal Medicine, Aichi Medical University School of Medicine, Nagakute, Japan; ^2^Division of Endocrinology and Metabolism, Department of Internal Medicine, Fujita Health University School of Medicine, Toyoake, Aichi, Japan; ^3^Department of Internal Medicine, Aichi Gakuin University School of Dentistry, Nagoya, Japan; ^4^Department of Medicine, Aichi Gakuin University School of Pharmacy, Nagoya, Japan

## Abstract

**Objective:**

Diabetic polyneuropathy (DPN) is one of the most prevalent diabetic complications. We previously demonstrated that exendin-4 (Ex4), a glucagon-like peptide-1 receptor agonist (GLP-1RA), has beneficial effects in animal models of DPN. We hypothesized that GLP-1 signaling would protect neurons of the peripheral nervous system from oxidative insult in DPN. Here, the therapeutic potential of GLP-1RAs on DPN was investigated in depth using the cellular oxidative insult model applied to the dorsal root ganglion (DRG) neuronal cell line.

**Research Design and Methods:**

Immortalized DRG neuronal 50B11 cells were cultured with and without hydrogen peroxide in the presence or absence of Ex4 or GLP-1(7-37). Cytotoxicity and viability were determined using a lactate dehydrogenase assay and MTS (3-(4,5-dimethylthiazol-2-yl)-5-(3-carboxymethoxyphenyl)-2-(4-sulfophenyl)-2H-tetrazolium inner salt), respectively. Antioxidant enzyme activity was evaluated using a superoxide dismutase assay. Alteration of neuronal characteristics of 50B11 cells induced by GLP-1RAs was evaluated with immunocytochemistry utilizing antibodies for transient receptor potential vanilloid subfamily member 1, substance P, and calcitonin gene-related peptide. Cell proliferation and apoptosis were also examined by ethynyl deoxyuridine incorporation assay and APOPercentage dye, respectively. The neurite projection ratio induced by treatment with GLP-1RAs was counted. Intracellular activation of adenylate cyclase/cyclic adenosine monophosphate (cAMP) signaling was also quantified after treatment with GLP-1RAs.

**Results:**

Neither Ex4 nor GLP-1(7-37) demonstrated cytotoxicity in the cells. An MTS assay revealed that GLP-1RAs amended impaired cell viability induced by oxidative insult in 50B11 cells. GLP-1RAs activated superoxide dismutase. GLP-1RAs induced no alteration of the distribution pattern in neuronal markers. Ex4 rescued the cells from oxidative insult-induced apoptosis. GLP-1RAs suppressed proliferation and promoted neurite projections. No GLP-1RAs induced an accumulation of cAMP.

**Conclusions:**

Our findings indicate that GLP-1RAs have neuroprotective potential which is achieved by their direct actions on DRG neurons. Beneficial effects of GLP-1RAs on DPN could be related to these direct actions on DRG neurons.

## 1. Introduction

Among many significant diabetic complications, diabetic polyneuropathy (DPN) is one of the most prevalent complications and causes nontraumatic amputations of lower limbs [1]. Due to the lack of therapies to address the etiology of neurodegeneration in the peripheral nervous system (PNS) of diabetic patients, glucose-lowering therapy is the only effective therapy to prevent the onset and progression of DPN [2]. In the current study, we investigated the beneficial effects of glucagon-like peptide-1 (GLP-1) signaling in neurons of the PNS using an *in vitro* model of DPN.

GLP-1, an incretin hormone which lowers blood glucose levels through enhancement of glucose-stimulated insulin secretion (GSIS), also has pleiotropic effects. In nervous systems, GLP-1 has a regulatory effect on food intake through the intermediary of the vagus nerve and the central nervous system (CNS) [3–7]. It is known that GLP-1 activates adenylate cyclase and employs cAMP as a second messenger to enhance GSIS in pancreatic beta cells [8, 9]. The cAMP signaling has been proven to stimulate neurite outgrowth [10, 11] and antagonize apoptosis of PNS neurons or PC12 cells [12]. In some kinds of nonneural cells including pancreatic beta cells and cardiomyocytes, antiapoptotic effects of GLP-1 receptor agonists (GLP-1RAs) have been also shown [13–16]. Additionally, it has been reported that activation of GLP-1 signaling modified cell fate and differentiation in pancreatic beta cells [17, 18]. GLP-1 signaling induced *in vivo* reprogramming of pancreatic exocrine cells into beta cells [17] and *in vitro* differentiation of human embryonic stem cells into insulin-producing cells [19].

Previously, we reported the beneficial effects of exendin-4 (Ex4) (also known as exenatide), a GLP-1RA, in the PNS of diabetic mice [20]. In that prior study, we indicated the improvement of DPN using an *in vivo* model but the mechanism of the favorable effects on the PNS has not yet been identified. Although we have proven that the elongation of neurite outgrowth using a tissue culture system of mouse dorsal root ganglion (DRG) was accelerated by supplementation of Ex4 or GLP-1, detailed effects of GLP-1RAs in the DRG should be still elucidated.

Among various mechanisms of pathogenesis in DPN, chronic inflammation followed by oxidative stress has been highlighted by several researchers [21, 22]. For instance, cyclooxygenase-2-deficient mice were protected from dysfunction of the PNS in experimental diabetes [23]. Given that oxidative stress due to various biological pathways, including chronic low-grade inflammation, has been suggested as a pathogenesis and a therapeutic target of DPN [21, 24, 25], we attempted to provide oxidative stress in our culture system. However, it remains to be clarified which factor is crucial in the pathology of DPN, e.g., glucotoxicity, insulin resistance, or lipotoxicity [21]. Therefore, we provided oxidative insult by hydrogen peroxide, which is a widely used oxidant in experimental settings and converts into the stronger oxidant hydroxyl radical, in the cell culture system of the DRG neuron cell line to reproduce DPN pathology in this study.

## 2. Materials and Methods

Unless noted otherwise, all reagents and materials were purchased from Thermo Fisher Scientific (Waltham, MA, USA).

### 2.1. Cell Culture

The DRG neuronal cell line (50B11) established and kindly provided by Dr. A. Höke (Johns Hopkins University, Baltimore, MD, USA) [26] was incubated at 37°C under 5% CO_2_ in media consisting of Neurobasal™ medium supplemented with 5% fetal bovine serum, 2 mM L-glutamine, and B-27 supplement. 50B11 cells were kept in uncoated plastic tissue culture dishes and regularly passaged once a week with a 1 : 10-1 : 20 split ratio. For each experiment as described in the sections, cells were treated with Ex4 (0.1 nM, 1 nM, 10 nM, and 100 nM), human GLP-1(7-37) (1 nM, 10 nM), or 10 *μ*M forskolin. Oxidative insult was induced by hydrogen peroxide (0.01 mM, 0.05 mM, and 0.1 mM).

### 2.2. Cell Cytotoxicity Assay

Cells were seeded into 96-well plates at a density of 1 × 10^4^ cells/well in 100 *μ*l medium. Cell cytotoxicity was assessed using lactate dehydrogenase (LDH) assay (Cytotoxicity LDH Assay Kit-WST, Dojindo Laboratories, Mashiki, Japan) following the manufacturer's instructions. The absorbance at 490 nm was measured on a microplate reader (VersaMax, Molecular Devices, Sunnyvale, CA, USA). Cytotoxicity was calculated by the following formula: cytotoxicity (%) = (sample OD–low control OD)/(high control OD–low control OD) × 100 (OD: optical density). Each OD value was calculated by subtracting the background value from each absorbance value.

### 2.3. Immunocytochemistry

To exclude the possibility of alteration in neuronal characteristics by GLP-1RAs which might induce a reprogramming of cell fate, the characteristics as a sensory neuronal cell were evaluated with the distribution of neuronal markers: transient receptor potential vanilloid subfamily member 1 (TRPV1), substance P, and calcitonin gene-related peptide (CGRP). After a 36-hour culture with or without 100 nM Ex4 or 10 nM GLP-1, DRG cells were fixed with 4% paraformaldehyde for 15 minutes. The cells were blocked with 1% bovine serum albumin, and the following primary antibodies were applied at 4°C overnight: rabbit polyclonal anti-TRPV1 antibody (1 : 200; Neuromics, Northfield, MN, USA), goat polyclonal anti-substance P antibody (1 : 200; Santa Cruz, Santa Cruz, CA, USA), and goat polyclonal anti-CGRP antibody (1 : 200; Santa Cruz). After washing, the following secondary antibodies were loaded for 1 hour at room temperature in a dark box: Alexa Fluor™ 594-coupled goat anti-rabbit IgG antibody (1 : 500) or Alexa Fluor™ 488-coupled donkey anti-goat antibody (1 : 500). Images were captured by a charge-coupled device (CCD) camera using a fluorescence microscope (IX73, Olympus Optical, Tokyo, Japan).

### 2.4. Cell Viability Assay

To elucidate the effects of GLP-1RAs in DRG neurons under oxidative stress, cell viability of DRG neurons cultured with or without hydrogen peroxide in the presence or absence of GLP-1RAs was assessed. A 3-(4,5-dimethylthiazol-2-yl)-5-(3-carboxymethoxyphenenyl)-2-(4-sulfophenyl)-2H-tetrazolium inner salt (MTS) assay, which correlated mitochondrial activity, was employed to measure cell viability in DRG neurons. Cells were seeded into 96-well plates at a density of 1 × 10^4^ cells/well in 100 *μ*l medium. Cell viability was determined 24 hours after treatment using the CellTiter96™ AQueous One Solution Cell Proliferation Assay (Promega Corporation, Madison, WI, USA), which employed tetrazolium compound MTS, according to the manufacturer's protocol. The absorbance at 490 nm was measured on a microplate reader (VersaMax).

### 2.5. Superoxide Dismutase- (SOD-) Like Activity

To evaluate antioxidant activity, SOD-like activity was measured using an SOD-like assay kit (Dojindo Inc., Kumamoto, Japan) according to the manufacturer's instructions [27]. Equal amounts of protein, as determined using a bicinchoninic acid protein assay (Wako Pure Chemical Inc., Osaka, Japan), were applied. Cells were seeded into 96-well plates at a density of 1 × 10^4^ cells/well in 100 *μ*l medium. After 24 hours, cells were supplemented with GLP-1RAs (10 nM GLP-1, 100 nM Ex4) or left untouched. After 12 hours of treatment with/without GLP-1RAs, the media were replaced with media containing 0.1 mM hydrogen peroxide. SOD-like activity was determined 30 minutes after the exposure with hydrogen peroxide.

### 2.6. Apoptosis Assay

For the apoptosis assay, 50B11 cells were seeded into 24-well plates at a density of 5 × 10^4^ cells/well. Apoptosis was induced by 0.1 mM hydrogen peroxide. The degree of apoptosis was assessed using the APOPercentage assay (Biocolor, Belfast, Northern Ireland, UK), which was performed according to the manufacturer's instructions. The APOPercentage assay is a dye uptake assay, which stains only the apoptotic cells with a purple dye [28]. Apoptotic cells were assessed after a 3-hour exposure to hydrogen peroxide with or without GLP-1RAs (GLP-1, Ex4) and forskolin. Absorption was measured at 550 nm using a microplate reader (VersaMax).

### 2.7. Cell Proliferation Assay

An ethynyl deoxyuridine (EdU) incorporation assay was performed using the Click-iT Plus EdU Proliferation Kit (Life Technologies Inc., Gaithersburg, MD). Cells were treated with 10 *μ*M EdU for 24 hours, then harvested, and fixed with 4% paraformaldehyde for 20 minutes. For EdU detection, cells were incubated with Alexa Fluor™ 488 Azide for 15 minutes and then counter stained with 4′,6-diamidino-2-phenylindole (DAPI) [29, 30]. The rate of proliferating cells was determined by the number of EdU-incorporating cells divided by that of DAPI-positive cells.

### 2.8. Neurite Outgrowth Assay in 50B11 Cells

As it has been verified that the 50B11 neuronal cell line can elongate neurites by stimulation with forskolin, the neurite outgrowth induced by GLP-1RAs was also examined to afford collateral evidence of the neuroregenerative ability in DRG neurons. 50B11 cells were plated into 6-well plates at a density of 1 × 10^4^ cells/well. Twenty-four hours after the passage of the cells, cells were unexposed or exposed to the indicated compounds for 24 h. Images of the cells were captured by a contrast-phase microscope equipped with a CCD camera and counted for neurite outgrowth which was defined as a process equal to or greater than cell bodies in length [31].

### 2.9. Cyclic Adenosine Monophosphate (cAMP) Assay

Cellular cAMP production was measured using an enzyme immunoassay kit (Cayman Chemical, Ann Arbor, MI, USA) [32, 33]. Cells were seeded into 6-well plates at a density of 5 × 10^5^ cells/well. The media were aspirated 20 or 120 minutes after exposure to test substances, and 250 *μ*l of 0.1 N HCl was introduced. After 20 minutes incubation at room temperature, cells were scraped and centrifuged. The supernatants were stored at -80°C until the time of measurement. For the experiment with 120-minute exposure to test substances, the medium contained 0.5 mM 3-isobutyl-1-methyl xanthine (IBMX), a phosphodiesterase inhibitor, to inhibit cAMP degradation.

### 2.10. Statistical Analysis

All the group values were expressed as means ± standard deviation. Data are representative of at least three independent experiments. The normality of distribution was tested by the Kolmogorov-Smirnov test using R version 3.4.3 (http://www.r-project.org/, Vienna, Austria,). Statistical analyses were made by Student's *t*-test or one-way ANOVA with the Bonferroni correction for multiple comparisons using StatView version 5.0 (SAS Institute, Cary, NC). The threshold of statistical significance was taken as a value of *p* < 0.05. All analyses were performed by personnel unaware of the identities of culture conditions.

## 3. Results

### 3.1. No Cytotoxicity Was Introduced by GLP-1RAs in DRG Neurons

**Figure 1:**
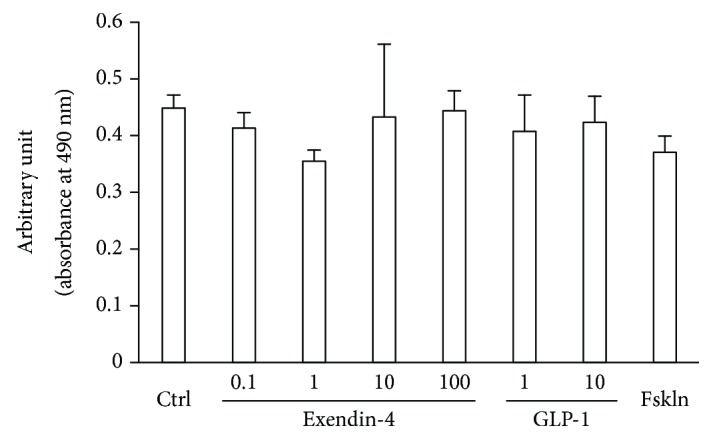
Cell cytotoxicity of GLP-1 receptor agonists (GLP-1RAs) in dorsal root ganglion (DRG) neurons. Cytotoxicity was determined 24 hours after treatment with GLP-1RAs or forskolin using LDH assay. No significant difference was detected between neurons treated with GLP-1RAs or forskolin and those without treatment (control). Concentrations of GLP-1RAs; exendin-4: 0.1, 1, 10, and 100 nM; GLP-1: 1, 10 nM. Ctrl: control; Fskln: forskolin; error bar: standard deviation. *n* = 3 in each group.

There was no significant cytotoxicity induced after 24 hour exposure to Ex4 (0.1 mM, 1 nM, 10 nM, or 100 nM) or GLP-1 (1 nM, 10 nM) (absorbance at 490 nm: control 0.449 ± 0.023, 0.1 nM Ex4 0.414 ± 0.027, 1 nM Ex4 0.355 ± 0.020, 10 nM Ex4 0.433 ± 0.129, 100 nM Ex4 0.444 ± 0.034, 1 nM GLP-1 0.408 ± 0.064, and 10 nM GLP-1 0.424 ± 0.046) (Figure 1). Neurons were also exposed to an adenylate cyclase activator, forskolin. The treatment with 10 *μ*M forskolin did not induce any significant difference in cytotoxicity (10 *μ*M forskolin 0.371 ± 0.029).

### 3.2. Sensory Neuronal Characteristics in Protein Marker Expressions Were Not Affected by GLP-1RAs

**Figure 2:**
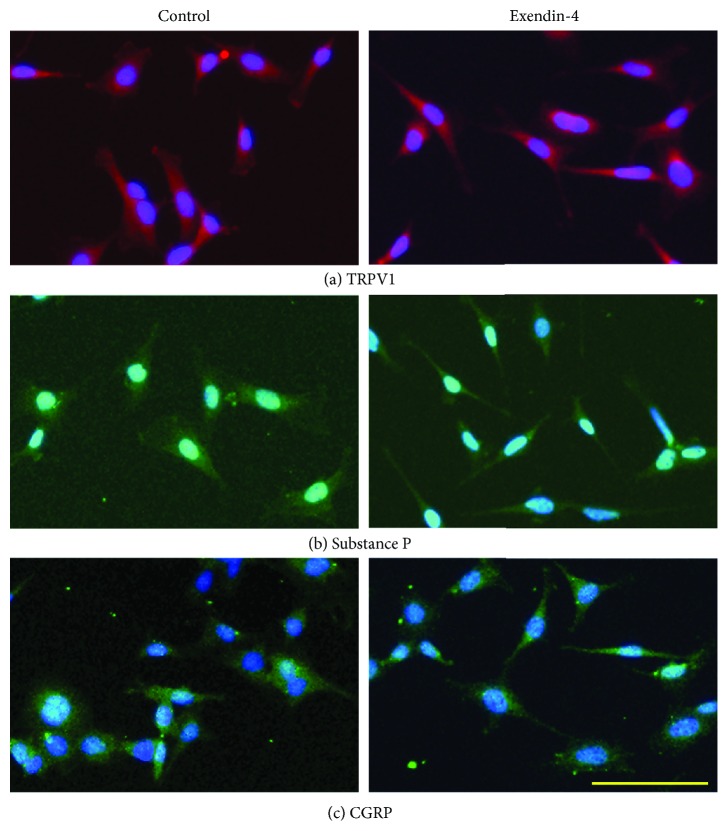
Distribution of sensory neuronal markers in the dorsal root ganglion (DRG) neuron cell line treated with exendin-4. Pictures on the left side are neurons without any treatment. Pictures on the right side are neurons treated with 100 nM exendin-4 for 36 hours. TRPV1: red (a), substance P: green (b), CGRP: green: DAPI (c), scale 100 *μ*m.

Ex4 or GLP-1 (data not shown) induced no evident changes in the distribution pattern of these sensory neuronal markers compared with neurons without those treatments (Figure 2).

### 3.3. Cell Viability Was Enhanced in DRG Neurons Cultured with GLP-1RAs

**Figure 3:**
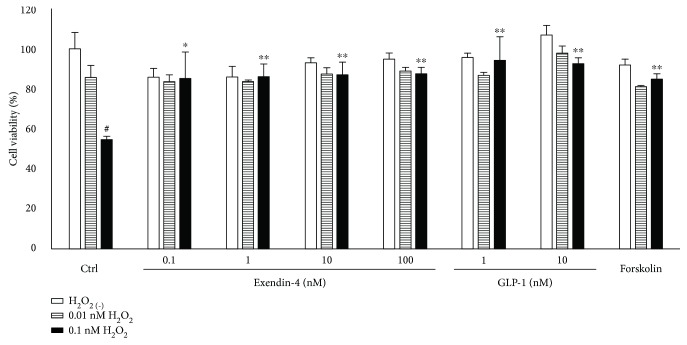
Cell viability in dorsal root ganglion (DRG) neurons treated with GLP-1 receptor agonists. Cell viability was quantified using 3-(4,5-dimethylthiazol-2-yl)-5-(3-carboxymethoxyphenenyl)-2-(4-sulfophenyl)-2H-tetrazolium inner salt (MTS). Although hydrogen peroxide (H_2_O_2_) significantly decreased the cell viability of DRG neurons, GLP-1 receptor agonists, exendin-4 and GLP-1, and forskolin, an activator of adenylate cyclase, prevent the reduction of cell viability induced by H_2_O_2_. White bar: no supplementation of H_2_O_2_; hatched bar: 0.01 mM H_2_O_2_; filled bar: 0.1 mM H_2_O_2_; Ctrl: control; H_2_O_2_: hydrogen peroxide; ^#^ *p* < 0.01 versus control without H_2_O_2_; ^∗^ *p* < 0.05 versus control with 0.1 mM H_2_O_2_; ^∗∗^ *p* < 0.005 versus control with 0.1 mM H_2_O_2_; error bar: standard deviation. *n* = 3 in each group.

The cell viability of DRG neurons treated with 0.1 mM hydrogen peroxide for 4 hours was significantly decreased compared with that of cells cultured with no hydrogen peroxide (control 100 ± 8.1%, 0.1 mM hydrogen peroxide 54.3 ± 2.1, *p* < 0.01) (Figure 3). However, the treatment with Ex4 or GLP-1 significantly ameliorated cell viability compared with cells with no treatment (0.1 nM Ex4 85.1 ± 13.3, 1 nM Ex4 86.0 ± 6.4, 10 nM Ex4 86.9 ± 6.5, 100 nM Ex4 87.5 ± 3.2, 1 nM GLP-1 94.3 ± 11.7, and 10 nM GLP-1 92.6 ± 2.9). The supplementation with 10 *μ*M forskolin also inhibited the decrease of cell viability (84.5 ± 2.6, *p* < 0.005).

### 3.4. SOD-Like Activity Increased in the Sensory Neurons Supplemented with GLP-1RAs

**Figure 4:**
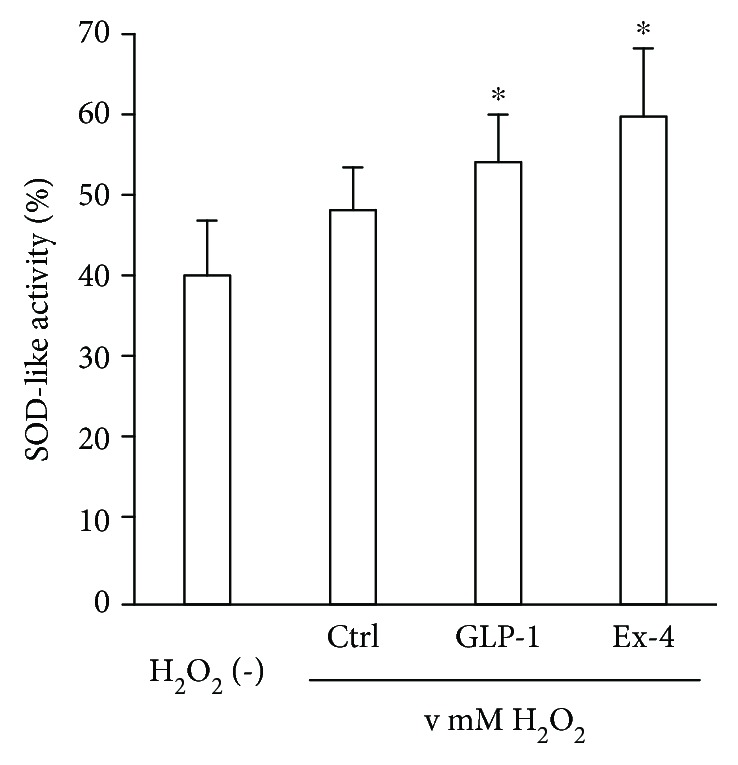
Superoxide dismutase- (SOD-) like activity in dorsal root ganglion (DRG) neurons treated with glucagon-like peptide-1 (GLP-1) receptor agonists. Oxidative insult induced by 30-minute treatment with 0.1 mM hydrogen peroxide increased SOD-like activity in the neurons supplemented with 10 nM GLP-1 or 100 nM exendin-4. ^∗^ *p* < 0.001 versus no treated cell with H_2_O_2_. H_2_O_2_: hydrogen peroxide; Ctrl: control; GLP-1: cells supplemented with 10 nM GLP-1; Ex-4: cells supplemented with 100 nM exendin-4. Error bar means standard deviation. *n* = 6 or 7 in each group.

Following exposure to oxidative insult with hydrogen peroxide, SOD-like activity increased in neurons supplemented with GLP-1 or Ex4 (cells with no hydrogen peroxide 40.4 ± 6.7%, 10 nM GLP-1 with 0.1 mM hydrogen peroxide 54.3 ± 5.8, and 100 nM Ex4 with 0.1 mM hydrogen peroxide 59.9 ± 8.4, *p* < 0.001 versus cells with no hydrogen peroxide in each GLP-1RA-supplemented group) (Figure 4).

### 3.5. Apoptosis Was Prevented in the Neurons Supplemented with Ex4

**Figure 5:**
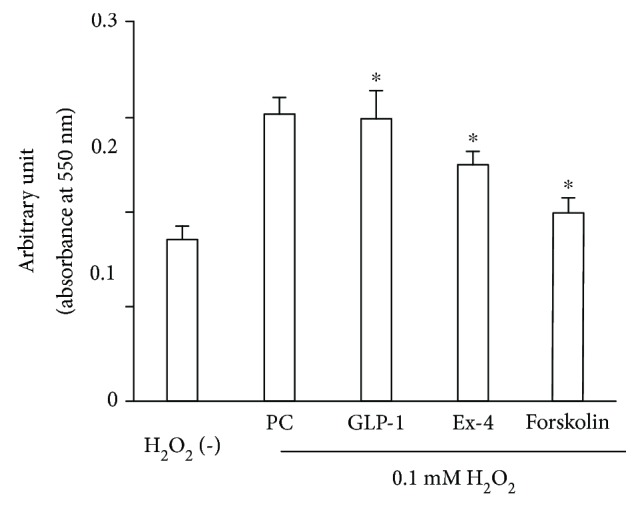
Apoptosis in dorsal root ganglion (DRG) neurons treated with exendin-4. Apoptosis induced by 3-hour treatment with 0.1 mM hydrogen peroxide was partially inhibited in the neurons supplemented with 100 nM exendin-4 or 10 *μ*M forskolin. ^∗^ *p* < 0.05 versus control; H_2_O_2_: hydrogen peroxide; PC: positive control of apoptosis; GLP-1: glucagon-like peptide-1; Ex-4: exendin-4. Error bar means standard deviation. *n* = 8 in each group.

Apoptosis evoked by 0.1 mM hydrogen peroxide was detected using the APOPercentage assay (Figure 5). The degree of apoptosis was significantly decreased in the neurons supplemented with 100 nM Ex4 (absorbance at 550 nm: control 0.304 ± 0.017, 100 nM Ex4 0.250 ± 0.014, *p* < 0.0001) and 10 *μ*M forskolin (0.199 ± 0.016, *p* < 0.0001). However, GLP-1 produced no significant change in the apoptosis assay (0.299 ± 0.03, *p* = 0.623).

### 3.6. Cell Proliferation Was Suppressed by GLP-1RAs

**Figure 6:**
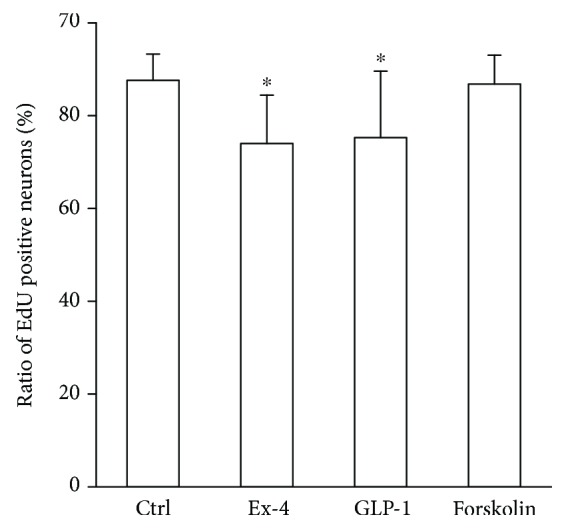
Proliferation rate of dorsal root ganglion (DRG) neurons treated with GLP-1 receptor agonists. Proliferation rate assessed by EdU assay revealed that both GLP-1 receptor agonists, exendin-4 and GLP-1, suppressed proliferation of DRG neurons. Ctrl: control; Ex-4: cells supplemented with 100 nM exendin-4; GLP-1: cells supplemented with 10 nM GLP-1; ^∗^ *p* < 0.05 versus control; error bar: standard deviation. *n* = 9 in each group.

The EdU incorporation assay revealed a decrease of proliferation rate of neurons cultured with 10 nM GLP-1 or 100 nM Ex4 (control 87.7%±5.6%, GLP-1 75.5 ± 10.4, and Ex4 74.1 ± 14.4) (Figure 6). However, forskolin had no significant effect on the proliferation rate (forskolin: 86.9 ± 6.2).

### 3.7. Neurite Outgrowth Was Induced with GLP-1RAs

**Figure 7:**
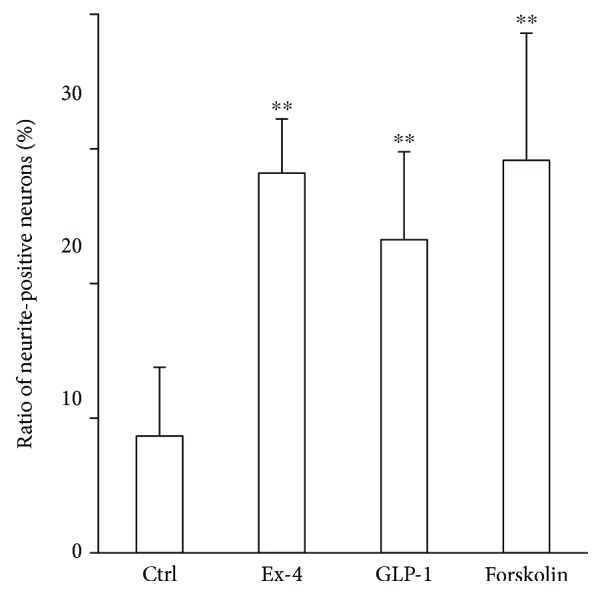
Neurite outgrowth of dorsal root ganglion (DRG) neurons. The ratio of neurite-positive neurons increased in cells supplemented with GLP-1 receptor agonists, exendin-4 and GLP-1, as well as cells which were supplemented with forskolin. Ctrl: control; Ex-4: cells supplemented with 100 nM exendin-4; GLP-1: cells supplemented with 10 nM GLP-1; forskolin: cells supplemented with 10 nM forskolin; ^∗∗^ *p* < 0.001 versus control; error bar: standard deviation. *n* = 9 or 15 in each group.

The percentage of neurons with neurite(s) increased in the neurons cultured with Ex4 or GLP-1 compared with the control (control 8.7%±5.1%, 100 nM Ex4 28.2 ± 4.0, and 10 nM GLP-1 23.3 ± 6.5, *p* < 0.0001 for both cases versus control) (Figure 7).

### 3.8. The Adenylate Cyclase/cAMP Pathway Was Not Activated by GLP-1RAs in DRG Neurons

**Figure 8:**
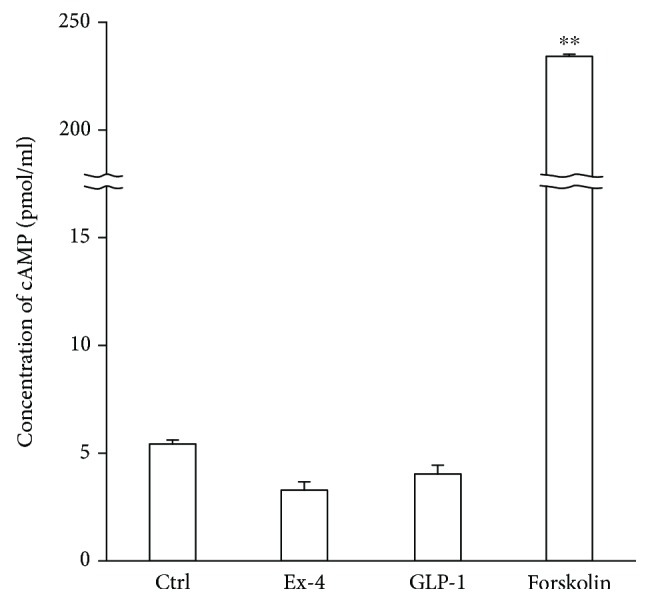
Intracellular cyclic adenylate monophosphate (cAMP) accumulation in neurons treated with GLP-1 receptor agonists. The cAMP accumulation was measured 20 minutes after exposure to 100 nM exendin-4, 10 nM GLP-1, or 10 *μ*M forskolin. Both GLP-1 receptor agonists, exendin-4 and GLP-1, provoked no significant cAMP accumulation. Ex-4: cells supplemented with 100 nM exendin-4; ^∗∗^ *p* < 0.001 versus control; error bar: standard deviation. *n* = 5 or 6 in each group.

Cyclic AMP levels after stimulation with GLP-1RAs and forskolin were determined. After 20 minutes of stimulation with 10 *μ*M forskolin, cAMP had accumulated in the neurons (control: 5.3 ± 0.3 pmol/ml, 10 *μ*M forskolin: 234.5 ± 6.3, *p* < 0.0001) (Figure 8). However, no accumulation of cAMP was detected in the neurons treated with Ex4 and GLP-1 (10 nM GLP-1: 3.3 ± 0.4, 100 nM Ex4: 4.0 ± 0.4). Longer exposure to GLP-1RAs supplemented with a phosphodiesterase inhibitor also generated no significant cAMP accumulation (Supplemental figure available here).

## 4. Discussion

In this decade, drug development targeting GLP-1 signaling has been considered as a prospective therapy of type 2 diabetes. A novel GLP-1RA semaglutide which can be orally administered would accelerate popularization of GLP-1RAs in clinical settings [34]. Furthermore, the neuroprotective effects of Ex4 have been already proven in one clinical trial of Parkinson's disease [35]. Therefore, if the neuroprotective effects of GLP-1RAs are accepted amongst the scientific community, a drug repositioning strategy of GLP-1RAs targeting other diseases will be promising, especially in diabetic complications including DPN.

In the current study, we investigated the neuroprotective effects of GLP-1RAs in the DRG neuronal cell line. First, we examined the neurotoxicity of GLP-1RAs in the DRG neurons. Second, we examined the effect of GLP-1RA on cell viability, antioxidant enzyme activity, and apoptosis in the DRG neurons. We confirmed enhanced cell viability, increased activity of antioxidant enzyme SOD, and inhibition of apoptosis with GLP-1RA supplementation. We then demonstrated that treatment with GLP-1RAs reduced cell proliferation and promoted neurite outgrowth of DRG neurons. Although these significant changes were seemed to be evoked by activation of the adenylate cyclase/cAMP pathway, no evident accumulation of intracellular cAMP was generated by stimuli with GLP-1RAs.

GLP-1RAs have previously been shown to promote neurite outgrowth in PC12 cells, a rat pheochromocytoma cell type [36, 37]. However, no report has investigated the direct pharmacological function of GLP-1RAs in the cells of the PNS, e.g. DRG neurons, Schwann cells, vascular endothelial cells in peripheral nerves. Some research studies, including our previous study, have already reported *in vivo* beneficial effects of GLP-1RAs in the disorders of the PNS [20, 38]. The current study would support these beneficial effects through verification of the direct effects of GLP-1RAs on DRG neurons.

A number of DPN pathogenesis mechanisms have been postulated in experimental studies, including the polyol pathway, advanced glycation end products, poly ADP-ribose polymerase, the protein kinase C pathway, and oxidative stress [39, 40]. In the current study, we chose oxidative stress to represent an *in vitro* DPN model. To verify the novel *in vitro* experimental system for investigation of DPN, we confirmed the characteristics of a 50B11 cell line as DRG neurons and induced oxidative insult on the cell line. After the confirmation of no cytotoxicity of GLP-1RAs and forskolin in 50B11, we evaluated the neuronal characteristics of the cells. The markers of a primary sensory neuron including TRPV1, substance P, and CGRP were expressed in 50B11 even after the treatment with GLP-1RAs. Furthermore, we successfully performed the neurite outgrowth assay, which is accepted as one of the crucial neuronal assays in a sympathetic-like neuron cell line PC12 [31]. As oxidative stress is one of the primary factors according to the prevailing views of DPN pathogenesis [39], we attempted to produce the pathogenesis utilizing hydrogen peroxide in the neuronal cell culture. Although, in clinical settings, several factors including dyslipidemia, hyperglycemia, hypertension, and smoking are considered to be risk factors of DPN [41], the significance of each oxidation mechanism derived from glucose, proteins, or lipids is unclear in the pathogenesis of DPN. Therefore, we utilized hydrogen peroxide, which is considered to be one of the most important reactive oxygen species because it crosses membranes and yields hydroxyl radicals via Fenton reaction in cells [42], as an oxidative insult-mimicking oxidative stress in DPN. As a result, hydrogen peroxide provoked an increase of antioxidant SOD in 50B11 cells. These experiments verified our experimental system as a novel approach to investigate DPN.

However, we must recognize some limitations of our study. As it is known that the incretin/adenylate cyclase/cAMP pathway is critical for insulin secretion in pancreatic beta cells [43] and neuroprotective effect in the CNS neurons [9], we compared pharmacological effects of GLP-1RAs with those of forskolin, an activator of adenylate cyclase, in DRG neurons. We proved the antiapoptotic effect of Ex4 and forskolin and the decrease of cell proliferation by GLP-1RAs. These findings were consistent with the previous report in which liraglutide, another GLP-1RA, potentiated cell viability and prevented apoptosis via cAMP signaling in SH-SY5Y neuroblastoma cells [44]. Furthermore, neurite outgrowth was induced by GLP-1RAs and forskolin. Given that background, these changes appear to indicate the activation of intracellular adenylate cyclase/cAMP signaling by GLP-1RAs as well as forskolin. However, unexpectedly, cAMP accumulation was not evident in the neurons cultured with GLP-1RAs for 20 or 120 minutes. This unexpected finding could be caused by the experimental limitation that our cAMP measurement kit was able to examine only the endpoint accumulation of cAMP. The activation of adenylate cyclase induced by GLP-1RAs might be more transient than we expected. Therefore, in the future, we would like to measure cAMP accumulation utilizing a real-time detection system.

Furthermore, we should consider scrutinizing other signaling pathways which have been reported to be initiated by GLP-1RAs. It is known that p44/42 mitogen-activated protein kinase (also called ERK1/2) can be also activated by GLP-1 in pancreatic beta cells [45]. It is also shown that the antiapoptotic effect of GLP-1 is mediated by ERK1/2 activation in beta cells [46]. Therefore, the antiapoptotic effect shown in the current study might be mediated by activation of ERK1/2 signaling.

Another limitation is the immortalization of the neurons. As the DRG neuronal cell line 50B11 cells are immortalized neurons, the differences between nonproliferative neurons collected from mammalians and the genetically engineered neurons should be taken into account. It was reported that an activation of phosphoinositide-3-kinase (PI3K) induced by GLP-1 in the beta cell line accelerated mitosis of the cells [47]. However, in this study, EdU incorporation was decreased by administration of GLP-1RAs. To address this conflict, in the future, we would clarify the involvement of PI3K signaling in sensory neurons [45, 46, 48].

## 5. Conclusions

This study is the first report to investigate the neuroprotective effects of GLP-1RAs on DRG neurons. The beneficial effects of GLP-1RAs in DPN might be attributable to the direct neuroprotective effects of GLP-1RAs on DRG neurons through protection from cellular oxidative insult.

At the same time, we successfully verified the novel *in vitro* experimental system for investigation of DPN.

## Data Availability

The whole data used to support the findings of this study are available from the corresponding author upon request.

## Abbreviations

cAMP: Cyclic adenosine monophosphate
CCD: Charge-coupled device
CGRP: Calcitonin gene-related peptide
CNS: Central nervous system
DAPI: 4′,6-Diamidino-2-phenylindole
DRG: Dorsal root ganglion
DPN: Diabetic polyneuropathy
EdU: Ethynyl deoxyuridine
Ex4: Exendin-4
GLP-1: Glucagon-like peptide-1
GLP-1RA: GLP-1 receptor agonist
GSIS: Glucose-stimulated insulin secretion
IBMX: 3-Isobutyl-1-methyl xanthine
LDH: Lactate dehydrogenase
MTS: 3-(4,5-Dimethylthiazol-2-yl)-5-(3-carboxymethoxyphenenyl)-2-(4-sulfophenyl)-2H-tetrazolium inner salt
PNS: Peripheral nervous system
SOD: Superoxide dismutase
TRPV1: Transient receptor potential vanilloid subfamily member 1.

## Supplementary Materials

Supplementary Materials Supplemental figure: intracellular cyclic adenylate monophosphate (cAMP) accumulation in neurons treated with GLP-1 receptor agonists and a cAMP/cGMP-phosphodiesterase inhibitor.
