# Supplementary material for: Glucagon-Like Peptide-1 Receptor Agonist Protects Dorsal Root Ganglion Neurons against Oxidative Insult
Source: J Diabetes Res. 2019 Feb 21;2019:9426014. doi: 10.1155/2019/9426014 (PMC6408997; doi:10.1155/2019/9426014)
Supplement: Supplementary Materials — Supplemental figure: intracellular cyclic adenylate monophosphate (cAMP) accumulation in neurons treated with GLP-1 receptor agonists and a cAMP/cGMP-phosphodiesterase inhibitor. [file 9426014.f1.pdf]

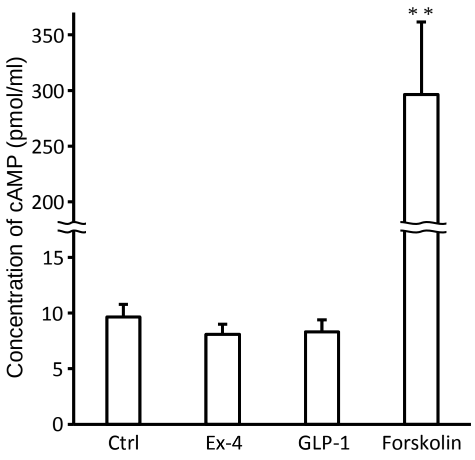

Supplemental figure. Intracellular cyclic adenylate monophosphate (cAMP) accumulation in neurons treated with GLP-1 receptor agonists and a cAMP/cGMP-phosphodiesterase inhibitor. The cAMP accumulation was measured 120 minutes after exposure to a cAMP/cGMP-phosphodiesterase inhibitor IBMX and 100 nM exendin-4, 10 nM GLP-1, or 10 μM forskolin. Both GLP-1 receptor agonists, exendin-4 and GLP-1, provoked no significant cAMP accumulation. Ex-4: cells supplemented with 100 nM exendin-4, **: $p < 0.001$ versus control. Error bar: standard deviation. n = 5 or 6 in each group.
